# The reliability of surgeons to avoid traumatic insertion of dental implants into high-risk regions: a panoramic radiograph study

**DOI:** 10.1186/s12903-020-01093-8

**Published:** 2020-04-06

**Authors:** Firas A. Jamil, Jamal A. Mohammed, Thair A. Hasan, Mohammed G. Rzoqi

**Affiliations:** 1 Department of Oral & Maxillofacial Surgery, Dental Teaching Hospital, College of Dentistry, University of Baghdad, Bab-Almoadham, P.O.Box 1417, Baghdad, Iraq; 2 Department of Biomedical Applications, Institute of Laser for Postgraduate Studies, University of Baghdad, Baghdad, Iraq

**Keywords:** Panoramic radiographs, Dental implant, Maxillary sinus, Mandibular canal, Inferior alveolar nerve

## Abstract

**Background:**

The posterior regions of the jaws usually represent a significant risk for implant surgery. A non-valid assessment of the available bone height may lead to either perforation of the maxillary sinus floor or encroachment of the inferior alveolar nerve and consequently to implant failure. This study aimed to evaluate the reliability of surgeon’s decision in appraising the appropriate implant length, in respect to vital anatomical structures, using panoramic radiographs.

**Methods:**

Only implants that are inserted in relation to the maxillary sinus (MS) or the mandibular canal (MC) were enrolled (first premolars [1P], second premolars [2P], first molars [1M], and second molars [2M]). All preoperative panoramic radiographs were evaluated under standard conditions. The postoperative estimation (under/over) was determined depending on the available bone height (ABH) measured from the apical end of the implant to the floor of the MS and the roof of the MC using cone beam computed tomography (CBCT). Any complication or side effect that associated with overestimated implants insertion was recorded.

**Results:**

The study sample included 73 patients (predominantly females) who had consecutively received 148 implants, of which 68 were inserted in the posterior maxilla and 80 in the posterior mandible. Underestimation was recorded in 93.2% of the measurements. The remaining bone height after implants insertion was < 2 mm in the majority of underestimated cases (73.9%); they were significantly (*P* < 0.01) more than sites with remaining bone ≥ 2 mm (26.1%). In the posterior mandible, overestimation was significantly higher than posterior maxilla. Five cases with transient paresthesia were reported in the mandibular overestimated implants.

**Conclusions:**

This study specified that surgeon’s choice of implants length, based on panoramic radiographs, was reliable regarding the incapability to insert implants with further length in the majority of underestimated cases, the low percent of overestimated measurements, and the minor associated complications.

## Background

With the increasing number of dental practitioners, the prospect of having complications with implant surgery has expected to be increased. Surgical complications and accidents do occur [1] and may cause damage of vital anatomical structures [2]. They may also result in infection, inflammation, and ultimately the loss of implants [3, 4]. Radiographic examination is an essential part of implant surgery and annually millions of these radiographs are taken for diagnosis and treatment [5]. To select an informative radiographic image, in addition to the patient’s history and clinical investigations, one should consider the diagnostic quality, region of interest, radiation dose, the financial burden, and accessibility. Radiographic misinterpretation can result in serious complications. These complications can involve damage to adjacent teeth and/or encroachment of vital structures, including the maxillary sinus and/or inferior alveolar nerve (IAN). The risk of penetration into the maxillary sinus was reported, as implants could be accidentally displaced into the sinus cavity [6]. However, the posterior mandible comprised the highest risk area during implant surgery. The incidence of IAN injury has been increased during implant surgery due to the proximity to the mandibular canal and mental foramen. Dental implants were found to be the most common etiological risk factor (56.3%) of nerve injury [7]. To avoid these complications, meticulous preoperative assessment is critical before insertion. Most implant surgeries can proceed uneventfully and fulfill functional and esthetic demands when proper diagnosis and treatment planning are implemented [8].

Prior to dental implant surgery, it is very important to assess the height of remaining alveolar bone in areas where implants are planned to be inserted, especially the posterior regions. The size of the maxillary sinus and its relationship with the upper teeth directly affect the surgical approach in this area [9]. Therefore, all oral surgeons have to be familiar with the anatomical limitations especially in the maxillary molar region. For these reasons, they should adopt appropriate surgical practices and inform patients about the possibility of any risk before operating [10]. In mandibular posterior teeth, the great majority present limited available bone between the root apex and the inferior alveolar canal (IAC). This stresses the need for careful attention during implant insertion in this area [8]. Otherwise, damage to the IAN may subsequently cause hemorrhage into the canal and/or neurosensory disturbances [7, 11]. This nerve was reported to be the most commonly injured nerve (64.4%), followed by the lingual nerve (28.8%) during oral surgery procedures [12]. According to the degree of injury, the altered sensation usually ranges from mild paresthesia to complete anesthesia [13]. Therefore, knowledge of accurate anatomical locations of the MC and mental foramen is an essential factor in the surgical management of any patient [14].

Numerous imaging techniques, including conventional radiographs (intraoral, tomography, panoramic radiographs, cephalometry), and computed tomography (CT) were proposed to localize the MC [15]. However, panoramic radiographs have a great role among them because of their reported benefits [16], and are still being used by the majority of surgeons as the only imaging modality in dental implant assessment [17]. They have been considered for a primary estimation to determine the bone height and implant-mandibular canal distance [18]. It was revealed that without using CT, panoramic radiographs were sufficient for measuring the height of alveolar bone for placement of implants in the mandibular posterior region, and there was little difference from cases that utilized linear or spiral CT [19]. In comparison with CT and other expensive tests, the panoramic image has a lower cost, rapid, greatly available with low radiation dose. Additionally, if metal prostheses are present, CT may produce streak artifacts. Furthermore, the patient should not move through a lengthy period of CT imaging [20]. However, panoramic radiograph like other methods has its own limitations such as high magnification, lower image clarity, distortion, low image resolution, and two-dimensional information without any sectional data [21, 22].

In implantology, panoramic views are commonly used for diagnostic purposes [23]. They had showed good visualization of the superior border of the MC, and it was convenient for treatment planning of dental implant surgery in the absence of other cross-sectional images (like CT), keeping in mind the dimensional changes and magnification factor [24]. As well, a linear tomogram and/or CT may be used. Although the frequency of application of these imaging techniques has been raised, pre-implant CT is not mandatory in all cases [25]. Even though the necessity for cross-sectional imaging has been intensely recommended, panoramic radiography is considered to be the standard radiographic examination for implant treatment planning since it has low radiation dose and provides valuable radiographic inspection [26, 27].

Although the CT evaluation can provide three-dimensional information [28], the cost and radiation dose of the procedure should be considered and outweighed by the value of expected information [29]. Even newly invented CBCT scans have a higher cost and cumulative radiation compared to panoramic radiographs [30]. The present research assesses the reliability of panoramic radiograph, as the only used imaging technique, in determining the ABH prior to implant placement in the maxillary and mandibular posterior sites as critical areas. It is the first study that specifically estimates the safety of assessment with panoramic radiographs in these high-risk regions. In addition to the evaluation of the soundness of implant length estimation (under/over), it also records the associated complications and their influence on the fate of the inserted implants, thus extending the knowledge in literature.

### Question in focus

Panoramic radiographs are still regarded as one of the most widely used imaging modality in implant surgery. This outlines the main goal of this study in answering the following question: is it safe for implantologist to assess the ABH depending only on panoramic radiographs?

## Methods

### Study design

The investigators planned and implemented a prospective study composed of patients who had undergone an implant surgery with different treatment modalities (conventional, immediate, piezoelectric site preparation, ridge split) at the Implant Unit of Dental Teaching Hospital between January 2010, and December 2016. The implant lengths used in this study (**Dentium®, Implantium®, Seoul, Korea**) were 8, 10, 12, and 14 mm.

Subjects eligible for study inclusion were those who had implants inserted at the maxillary and mandibular posterior regions. Any patient with implants that required to be installed at the anterior regions, implants that did not have any relation with the MS and MC, or radiographs presented with unclear findings were excluded from enrollment.

All the included patients have signed the informed consent regarding their agreement for the implant surgery. The whole study was approved by the institutional review board (Ethics Committee) of the College of Dentistry, University of Baghdad.

### Preoperative assessment

The implants selected for this study were assessed preoperatively and inserted by a single, skilled, and well-experienced oral and maxillofacial surgeon (TH), while the postoperative evaluation was performed by other surgeon (FJ) to prevent any bias. To obtain a standardized methodology, all of the evaluated panoramic radiographs were requested from the same unit (**Carestream Dental, CS 8100 Digital Panoramic System, New York, USA**). The preoperative assessment of ABH, taking into consideration the magnification rate, was determined from the alveolar crest to the superior border of the MC and from the crest of the bone to the inferior border of the MS using digital Vernier Caliper and LED x-ray film viewer. Regarding the MC, 2 mm were subtracted as a safety margin to determine the length of implant to be inserted.

### Postoperative assessment

After implants insertion, this level (2 mm from MC) was considered as a zero point from which the measurements were referred as either underestimated (positive value that was distant to 2 mm from the MC) or overestimated (negative value that was closer than 2 mm to the MC). Concerning the upper posterior region, the floor of the MS represented the borderline between underestimation (positive value that was distant to the floor of the MS) and overestimation (negative value that was inside the MS). The estimation (under/over) was determined depending on the distance measured from the apical end of implant to the floor of the MS and the roof of the MC using KODAK 9500 3D® CBCT (**Carestream Health, Inc., Marne-la-Vallée, France**). All patients were followed-up in the first year following implants insertion for any abnormal sign and/or symptom. Any complication associated with overestimated implants (inside the sinus or closer than 2 mm from the MC) was assessed and recorded. The validity of surgeon’s decision was depended on the amount of ABH in underestimated sites (< 2 mm / ≥ 2 mm), the percentage of overestimated implants insertion (low/high), and the adverse influence of reported complications (transient complications: ecchymosis, hematoma, hemorrhage, temporary paresthesia; permanent complication: persistent anesthesia).

### Statistical analysis

Statistical package of social science program (**SPSS version 24 for Windows, SPSS Inc., Chicago, IL, USA**) was used to analyze the data. Descriptive statistics (mean, range, frequency, minimum, maximum, percentage) and standard deviations (SD) were computed for each study variable. The independent sample T-test was used to determine whether there is a statistically significant difference between the means in two groups. Two proportions Z-test was utilized to compare the measured variables between maxilla and mandible in overestimated sites. The significance level was considered at *P*-value ≤0.05.

## Results

Eighty patients were available to be enrolled. Seven of them were excluded due to unclear anatomical findings of their panoramic radiographs. As a result, 73 patients (21 males and 52 females) with age ranged between 18 and 65 years (mean age = 42.1) were included in this study. A detailed description of the study sample including the total number of used implants with their lengths is presented in Table 1.

**Table 1:** Summary of study variables for the entire sample

Study variable	Descriptive statistics N (%)
Sample size (implants):	148 (100)
Posterior maxilla	68 (45.9)
Posterior mandible	80 (54.1)
Implant length (mm):	
8	32 (21.6)
10	61 (41.2)
12	52 (35.1)
14	3 (2.1)

N, number of occurrences

The findings of postoperative CBCT scans showed underestimation in 138 sites (93.2%); there was a highly significant difference between overestimation (10 sites; 6.8%) and underestimated readings (*P* < 0.01; Table 2).

**Table 2:** Descriptive statistics for the outcome variables

Variable	N	Premolars	Molars	Minimum (mm)	Maximum (mm)	Mean ± SD	*P* value
**Underestimated sites:**	**138**	**55**	**83**	0.2	6	1.58 ± 1.43	0.008^a,**^
Posterior maxilla Posterior mandible	67 71	37 18	30 53				
**Overestimated sites:**	**10**	**3**	**7**	−0.2	−1.6	0.36 ± 0.44	
Posterior maxilla Posterior mandible	1 9	1 2	0 7				

^a^By T-test (2-tailed)

^**^Highly significant

Regarding underestimation, the results showed non-significant difference (*P* = 0.256) between posterior maxilla (mean = 1.72 ± 1.5) and posterior mandible (mean = 1.45 ± 1.37). The ABH was < 2 mm in 102 sites (73.9%) of the underestimated distances, and they were significantly (*P* < 0.01) more than sites with ABH ≥ 2 mm (26.1%). The ranges of ABH in relation to different sites of underestimated implants are shown in Table 3.

**Table 3:** The relation between implant sites and remaining bone height after insertion in underestimated cases

Underestimated sites (***N*** = 138)		ABH N (% within implant site)		Total
		< 2 mm	≥ 2 mm	
**Posterior maxilla**	**1P**	7 (63.6)	4 (36.4)	**11**
	**2P**	18 (69.2)	8 (30.8)	**26**
	**1M**	19 (86.4)	3 (13.6)	**22**
	**2M**	6 (75)	2 (25)	**8**
**Posterior mandible**	**1P**	3 (75)	1 (25)	**4**
	**2P**	10 (71.4)	4 (28.6)	**14**
	**1M**	28 (70)	12 (30)	**40**
	**2M**	11 (84.6)	2 (15.4)	**13**
**Total**		**102**	**36**	**138**

*ABH* Available bone height after implants insertion

The overestimated implants were recorded at 1 site in the posterior maxilla and 9 sites in posterior mandible. Statistically, there was a highly significant difference (*P* = 0.00034) between the two regions. A detailed statistics of overestimated implants regarding the jaws and sites in relation to implant lengths are presented in Table 4.

**Table 4:** The percentages of different types of implant length that had caused overestimation

Implant length (mm)	Jaw	Site	Amount of overestimation (mm)	N	% of total
8	Mandible	1M	- 0.2	1	0.7
8	Mandible	1M	- 1.6	1	0.7
8	Mandible	2M	- 0.2	3	2
8	Mandible	2P	- 0.2	1	0.7
10	Maxilla	2P	- 0.4	1	0.7
10	Mandible	1P	- 0.2	1	0.7
12	Mandible	2M	- 0.2	2	1.3
Total				**10**	**6.8**

The reported complications revealed 5 cases with transient paresthesia, ranged 5 to 21 days, in the mandibular overestimated implants. Inferior alveolar nerve injury (direct trauma from implant) was not reported in this study.

## Discussion

In order to insert an implant with further length, we need at least 2 mm of ABH as the length of the used implant system is increased by 2 mm for each successive unit (Table 1). In the present study, the majority of measurements (93.2%) were underestimated with a non-statistically significant difference between posterior maxilla and mandible (*P* > 0.05). They were significantly more than overestimated sites (Table 2). However, this high percentage of underestimation did not mean a high percentage of short implants, where the ABH in most of the underestimated sites (73.9%) was less than 2 mm. As a result, no additional length of dental implant can be used. Akdeniz et al. [31] compared bone height and density measurements of implant recipient sites by panoramic radiography and computed tomography. Their results suggested that panoramic radiography significantly underestimated the bone height compared with CT. Another study conducted by Zarch et al. [16], which aimed to evaluate the accuracy of panoramic radiography in linear measurements of the jaws, showed underestimation in 83% of the measurements. These findings were consistent with the outcomes of this study. Our results contrast those of Fortin et al. [32] and others [33, 34] who concluded that distances were overestimated on the panoramic radiographs.

In both posterior regions, most of the underestimated sites presented with available bone (after insertion) of less than 2 mm (Table 3). Regarding maxilla, the most reliable choice of implant length was at the 1M sites (86.4%), followed by the 2M (75%), then the 2P (69.2%), and finally the 1P (63.6%). However, the mandibular posterior region showed different results from the maxilla where the 2M sites were the most reliable one (84.6%), followed by 1P (75%), then the 2P (71.4%), and the 1M (70%). Therefore, a proper attention should be given for the least reliable sites in a specific region.

Ten implants with overestimation were recorded in our study; there was an important difference between posterior maxilla and mandible (*P* < 0.01). The higher percentage was at the mandibular posterior region (9 sites) with just 1 site at the posterior maxilla presented at the 2P site (Table 4). Interestingly, the mandibular 2M had more tendency for overestimation (5 out of 9 sites), followed by the mandibular 1M (2 out of 9 sites), while both mandibular premolars (1P and 2P) had an equal existence (1 site for each). Therefore, the risk of IAN injury is more expected to occur at the 2M area. These results are in agreement with those of Lin et al. [11] who concluded that mandibular 2M presented highest risk for IAN injury (3.82 times) than other tooth type.

Little evidences existed concerning the minimum safe distance to guard the IAN during bone drilling; there was an obvious controversy about this subject. Sammartino et al. [35] suggested a distance of 1 mm between the implant and mandibular canal to prevent damage to the underlying nerve. For clinical safety, the authors recommended an additional 0.5 mm as a cushion. Accordingly, a 1.5 mm of minimal distance should be planned to avoid potential nerve injury. Hartmann et al. [36] concluded that sensory disturbances of the IAN could be avoided by keeping an average safety zone of 2.65 mm between implant and nerve. The results of our study showed that even when implants are inserted closer than 2 mm to the IAN (Table 4), there was no any permanent damage to the nerve as all of the reported complications (5 cases of paresthesia) were transient and cured spontaneously without any intervention. These results were in accordance with Tufekcioglu et al. [37] who suggested that if the operator can avoid thermal, pressure, and traumatic damage to the IAN, the implants can be placed closer than 2 mm to the inferior alveolar canal. Furthermore, the authors concluded that when 2 mm was considered as a safety level, the distance from implants to the IAC did not yield any statistical difference regarding postoperative neurosensory complications.

## Conclusions

Regarding posterior regions of the jaws, there is a tendency toward underestimation in panoramic radiographs, which is better than overestimation. Measurements at the maxillary 1M and 2M sites were more often underestimated by less than 2 mm than the measurements of the maxillary 1P and 2P sites. Measurements of the mandibular 2M site were more often underestimated by less than 2 mm than measurements of the other mandibular sites. Maxillary 1P and mandibular 1M sites presented the highest rate of underestimated measurements of ≥ 2 mm than the other sites. Overestimated measurements were more often seen in the mandible in comparison to the maxilla. Moreover, implants inserted closer than 2 mm to the inferior alveolar canal did not cause any permanent damage or injury to the IAN.

## Data Availability

The datasets used and/or analyzed during the current study are available from the corresponding author on reasonable request.

## Abbreviations

MS: Maxillary sinus
MC: Mandibular canal
1P: First premolars
2P: Second premolars
1M: First molars
2M: Second molars
ABH: Available bone height
CBCT: Cone beam computed tomography
IAN: Inferior alveolar nerve
IAC: Inferior alveolar canal
CT: Computed tomography
SD: Standard deviations
N: Number
